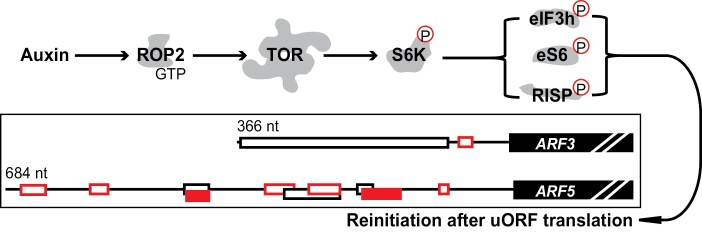# Do plants drive translation reinitiation to dodge nonsense-mediated decay?

**DOI:** 10.1093/jxb/erac444

**Published:** 2022-12-19

**Authors:** Yihan Dong, Lyubov A Ryabova

**Affiliations:** Institut de biologie moléculaire des plantes UPR2357 du CNRS, Université de Strasbourg, Strasbourg, France; Institut de biologie moléculaire des plantes UPR2357 du CNRS, Université de Strasbourg, Strasbourg, France

**Keywords:** NMD, post-transcriptional control, proteomics, mRNA degradation, translation reinitiation, UPFs

## Abstract

This article comments on:

**Cymerman MA, Saul H, Farhi R, Vexler K, Gottlieb D, Berezin I, Shaul O.** 2023. Plant transcripts with long or structured upstream open reading frames in the *NDL2* 5ʹ UTR can escape nonsense-mediated mRNA decay in a reinitiation-independent manner. Journal of Experimental Botany **74**, 91–103.

**In eukaryotes, a large proportion of mRNAs contains one or multiple upstream ORFs (uORFs) and their translation can cause a drastic reduction in main ORF translation. Furthermore, reinitiation-non-permissive uORFs can trigger degradation of their mRNAs via nonsense-mediated mRNA decay (NMD)—a process well documented in mammals. Cymerman *et al.* (2023) investigated whether the NMD pathway can target mRNAs with long and structured uORFs in Arabidopsis. They demonstrate that, in contrast to the observations in mammalian cells, these reinitiation-non-permissive uORFs do not trigger mRNA degradation by NMD.**

The primary cap-dependent mechanism of translation initiation operates mainly through linear scanning of mRNA leaders, where initiation occurs at the first 5’-AUG start codon in an appropriate initiation context. However, if this 5’-AUG codon is followed by a uORF, a fraction of the ribosomes that have completed translation of this uORF dissociates, resulting in a decrease in translation of the main downstream ORF (von Arnim *et al*., 2014; Schepetilnikov and Ryabova, 2018). Considering that up to 50% of eukaryotic mRNAs are loaded with one or more uORFs that can negatively regulate translation of the downstream main ORF, studying the mechanisms that control translation reinitiation and degradation of uORF-containing mRNAs (uORF-mRNAs) is of paramount importance.

Exported mRNAs normally go through a quality control step to reveal any abnormal features in the NMD pathway. This translation-dependent surveillance system detects mRNA defects that compromise the integrity of translation termination, and triggers the degradation of such aberrant transcripts, which may often have physiological consequences if translated. Up to 20% of endogenous transcripts, including functional protein-coding as well as non-coding RNAs, are NMD targets due to the presence of NMD-eliciting signatures such as a long 3ʹ-untranslated region (UTR; >1000 nt; Buhler *et al*., 2006), a premature termination codon (PTC) resulting from aberrant or alternative splicing events, or a long uORF (>30–60 codons) within the 5ʹ UTR (Karousis and Muhlemann, 2019).

## Mammalian NMD: RNA targets and protein factors

The classical NMD target is a termination codon positioned >50–55 nt upstream of the last exon–exon junction (EEJ). In such cases, the EEJ protein complex (EJC), located ~24 nt upstream of the EEJ, can initiate the assembly of NMD and RNA decay factors to degrade the mRNA (Lykke-Andersen and Jensen, 2015; Karousis and Muhlemann, 2019). However, if the PTC is close enough to the EEJ, translating or terminating ribosomes can physically remove the EJC protein complex and thus avoid NMD.

The early phase of NMD in eukaryotes includes PTC recognition and NMD complex formation. In mammals, the factor UPF1 (ATP-dependent RNA helicase up frameshift 1) is recruited through direct interaction with eukaryotic release factor 3 (eRF3), which is exposed on the ribosome during inefficient termination. UPF1 recruitment is assisted by UPF2 and UPF3, both of which are recruited by the EJC protein complex. The UPF1–UPF2–UPF3 complex promotes activation of UPF1 helicase activity. Phosphorylation of UPF1 by another NMD factor, SMG1 (SUPPRESSOR OF MORPHOLOGICAL DEFECTS ON GENITALIA 1), probably stops translation initiation events and starts recruitment of factors responsible for mRNA degradation (Shaul, 2015; Karousis and Muhlemann, 2019). In addition, several translation factors including the cap-binding complex (CBC), poly(A)-binding protein cytoplasmic 1 (PABC1), and eRFs1 and 3 can participate in NMD target recognition. A CBC component, eukaryotic translation initiation factor 4G (eIF4G), binds eRF3–PABC1, thereby circularizing the mRNA. However, a long 3’ UTR prevents formation of a complex between PABC1 and eRF3, which leads to inefficient termination and recycling of ribosomes, and thus activation of NMD (Fatscher *et al*., 2014). The late phases of NMD include translation repression and the degradation of mRNAs. At this step, SMG1 recruits the endonuclease SMG6 to cleave the mRNA. Further 3ʹ to 5ʹ mRNA decay is probably promoted by the exosome and followed by 5ʹ to 3ʹ degradation by exoribonuclease 1 (XRN1). To start the next round of NMD, the SMG5–SMG6 complex recruits protein phosphatase 2A to dephosphorylate and recycle UPF1, while the SMG8–SMG9 complex inactivates SMG1 kinase activity.

## When are uORFs inhibitory for translation of mRNAs?

NMD targets various mRNAs, including those harbouring one or more uORFs, resulting in highly variable outcomes at both transcript and protein levels. Noting the wide distribution of uORFs among eukaryotic mRNAs, uORF-containing transcripts would be a major group of NMD-targeted physiological mRNAs in eukaryotes if those uORFs do indeed trigger NMD. uORFs are positioned in mRNA 5ʹ UTRs upstream of their main protein-coding regions (Box 1) and usually function as repressors of main ORF translation. It is assumed that only a fraction of ribosomes that have completed uORF translation can resume scanning and reinitiate translation at a start codon further downstream. Reinitiation in eukaryotes occurs mainly if a uORF in a favourable initiation context is relatively short (encoding <20–30 amino acids). The critical parameter for efficiency of reinitiation is the short time taken for the ribosome to translate the uORF. Thus, the uORF may become non-permissive if it has a secondary structure that would be expected to cause ribosome pausing or if it is a further downstream uORF that would be initiated by the same ribosome that has already translated an upstream uORF. In mammals, translated uORFs that encode peptides longer than 30–50 amino acids are considered as reinitiation-non-permissive uORFs and thus can trigger NMD-dependent degradation of mRNAs (Romao *et al*., 2000; Inacio *et al*., 2004). Most of our knowledge of the NMD process to date comes from studies in mammalian systems. Puzzling points in plants include the facts that (i) high-throughput studies indicate that uORF-mRNAs have relatively high mRNA abundance in Arabidopsis at steady-state levels (Li and Liu, 2020); (ii) a significant fraction of uORF-mRNAs are not overexpressed in NMD mutants (Drechsel *et al*., 2013); and (iii) mRNAs that are significantly up-regulated in an *upf1-5* mutant contain long uORFs lacking suitable initiation context (and thus possibly not translated), indicating that uORF translation is not the most critical parameter activating NMD in Arabidopsis (Lukhovitskaya and Ryabova, 2019).

The recent study of Cymerman *et al*. (2023) addresses the key question of whether reinitiation-non-permissive uORFs characterized by long length and intense secondary structure can induce NMD in plants. To understand whether actively translated uORFs can induce NMD degradation, reporter genes with uORFs of varying lengths and secondary structures were used to compare mRNA reinitiation efficiency and degradation. The study was based on insertion of artificial uORFs into the 5’ UTR of the *NDL2* gene, which contains a native, reinitiation-competent, three amino acid uORF in a favourable initiation context. The authors asked whether extension of the three amino acid uORF to 13, 25, or 40 amino acids would alter the level of transcript degradation. Although uORF extension resulted in inhibition of translation of the main coding region in ascending order of uORF length, this did not result in transcript degradation. Another related point is that even greater extension of the native uORF to 50 or 70 amino acids, which abolishes reinitiation at the downstream reporter ORF, also did not cause transcript degradation. Similarly, the authors demonstrated that highly structured, and therefore reinitiation-non-permissive, uORFs can also escape NMD. These results have led to the suggestion that in plant cells, in contrast to the mammalian system, some transcripts with reinitiation-non-permissive uORFs do not undergo NMD, although the nature of such resistance remains unclear. Thus, in plants, translated uORFs may not trigger global transcript degradation, with only a subset of uORF-mRNAs being targeted. It has also been reported that CPuORFs (Box 1), which can completely prevent reinitiation in response to small metabolites, can trigger NMD in Arabidopsis (Uchiyama-Kadokura *et al*., 2014). Moreover, a uORF can also trigger NMD if it overlaps the main ORF for >17 nt (Kalyna *et al*., 2012).

## Upstream control of translation reinitiation: future research

Reinitiation in plants is facilitated by the key regulator of growth and development target of rapamycin (TOR) (Box 2) in response to nutrient and energy availability (Schepetilnikov and Ryabova, 2018). The small GTPase ROP2 has been identified as an upstream TOR effector that binds and activates TOR (Schepetilnikov *et al.*, 2017). The ROP2–TOR–S6K1 signalling axis attenuates the inhibitory effect of uORFs by enhancing plant reinitiation capacity through phosphorylation and activation of reinitiation-promoting factors (RPFs) that help the terminating ribosome to resume scanning and rapidly reacquire the ternary complex (eIF2–GTP–Met tRNA) and the 60S ribosomal subunit (Schepetilnikov *et al.*, 2013, 2017). In Arabidopsis, we have shown that, under conditions of TOR activation, levels of translation reinitiation of *ARF3* and *ARF5* can be increased several fold without affecting mRNA stability (Schepetilnikov *et al.*, 2017). Moreover, we assessed the content of endogenous *ARF3* and *ARF5* in polysomal and ribosome-less fractions in extracts isolated from wild-type or constitutively active ROP2 (CA-ROP2; highly active TOR) using sucrose gradient sedimentation (Schepetilnikov *et al.*, 2017). We found that ~70% of *ARF3* and *ARF5* mRNAs were present in the non-ribosomal fraction for the wild type, while ~60% of these mRNAs were associated with polysomes and ribosomes in CA-ROP2 extracts, despite total transcript levels being the same in both extracts. Thus, these cytoplasmic uORF- mRNAs are protected against degradation. Note that *ARF5* mRNA, which harbours multiple uORFs within its leader region, is not exposed for degradation in Arabidopsis extracts under contrasting TOR activation conditions.

Although the general NMD mechanism is conserved in plants (Shaul, 2015), several mammalian protein orthologues, such as SMG5, SMG8, SMG9, and SMG1, have not been found in Arabidopsis (Lloyd and Davies, 2013). Given that NMD-inducing features within uORF-mRNAs are not well defined, further work is clearly required to examine factors involved in stabilization of specific mRNAs. It would be interesting to investigate whether RPFs can play a role in putative stabilization of uORF-mRNAs. On the other hand, NMD is largely insensitive to plant transcripts with retained introns (Gohring *et al*., 2014), posing yet another question regarding the mechanism of NMD in plants. Thus, there is no doubt that this study by Cymerman *et al*. (2023) is an important step forward in understanding post-transcriptional control in plants, in particular the regulation of a large number of uORF-containing messages through reinitiation and NMD mechanisms.

**Box 1. Diverse types of uORFs**

After termination of translation, the 80S ribosome usually disassembles since it cannot resume scanning and reinitiate translation at a downstream ORF on the same mRNA. Reinitiation in mammals and plants occurs only if the first uORF is short, while a relatively long uORF (>60 codons) or a combination of several short uORFs can inhibit resumption of scanning and downstream main ORF initiation by post-terminating ribosomes and thus reduce mRNA translation efficiency by 30–80% (von Arnim *et al*., 2014). The AUG initiation context can influence uORF recognition: the optimal initiation context contains R (A or G) at position –3 and G at position +4 (RxxAUGG) as shown by filled red rectangles (Kozak, 1986). In plants, even a moderate context, missing either R or G at –3 or +4, respectively (open red rectangles), is recognized by ribosomes. A uORF in a weak initiation context lacking R and G (open black rectangles), or located close to the 5’ cap, can be bypassed by leaky scanning ribosomes (Schepetilnikov and Ryabova, 2018).

uORFs are classified by their inhibitory capacities and/or the evolutionary conservation of a peptide encoded by that uORF. uORFs that overlap the main ORF have the most significant inhibitory impact on mRNA translation. A small but rapidly increasing number of mRNAs have been identified as containing (usually) a single uORF (the initiation context is optimal) encoding an evolutionarily conserved peptide (CPuORF shown by the filled blue rectangle) with a few amino acid residues being critical for the uORF inhibitory effect (von Arnim *et al.*, 2014). Translation of CPuORF can trigger stalling of the translating ribosome at the CPuORF stop codon, leading to translation arrest and rapid mRNA degradation. Many uORFs that do not harbour CPuORFs are characterized by their differential ability to inhibit main ORF translation. In some cases, both non-CPuORFs and CPuORFs can control mRNA translation efficiency comprehensively. For example, *S*-adenosylmethionine decarboxylase (SAMDC) mRNA contains a single CPuORF, which inhibits SAMDC translation at high spermidine levels, while the inhibitory effect of other uORFs within the SAMDC mRNA leader can be overcome by translation reinitiation (Salazar-Díaz *et al*., 2021). Thus, translation of uORF-mRNAs can be controlled in a highly selective and specific manner.

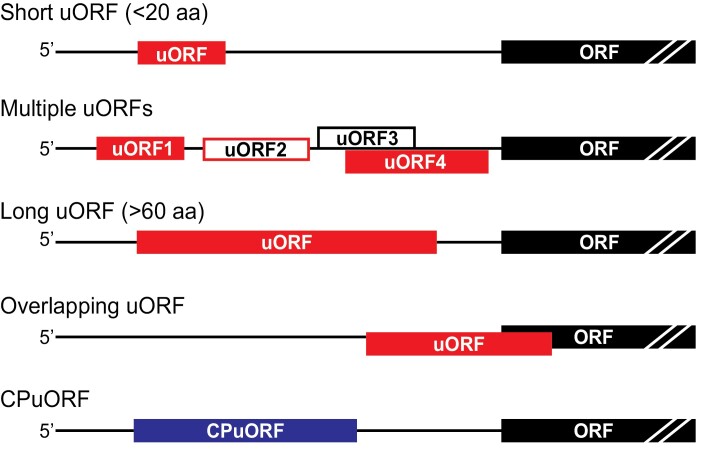

**Box 2. How TOR stimulates translation reinitiation of uORF-mRNAs**

TOR is a highly conserved serine/threonine protein kinase encoded by a single-copy gene in mammals and plants. In plants, TOR is significantly implicated in translation reinitiation of a multitude of mRNAs that harbour uORFs within their leader regions. In plants, an active TOR relay can overcome uORF repression by promoting reinitiation after uORF translation via a series of phosphorylation events, such as phosphorylation of eIF3 subunit h (eIF3h; Schepetilnikov *et al*., 2013), reinitiation supporting protein (RISP), and the 40S ribosomal protein S6 (rpS6; Mancera-Martínez *et al*., 2021). In Arabidopsis, a C-terminal truncation of eIF3h has no major effect on the first initiation event but almost abolishes mRNA reinitiation capacity. Indeed, h-less eIF3 can still interact with 40S and promote cap-dependent initiation events, indicating that eIF3h is a specific translation reinitiation factor in plants. Recent work also provides striking insight into the function of rpS6 in translation (Mancera-Martínez *et al*., 2021). Although rpS6 phosphorylation was not demonstrated to have a positive effect on global protein synthesis, its high phosphorylation status is important for reinitiation after uORF translation.

Many uORF-mRNAs are enriched in auxin signalling and polyamine metabolism. Auxin activates TOR via the GTP-bound ROP2 GTPase (Schepetilnikov *et al.*, 2017). Activated TOR stimulates translation of uORF-mRNAs, such as *ARF3* and *ARF5*, leading to positive feedback of auxin signalling (Schepetilnikov *et al*., 2013). A recent study also discovered that TOR senses spermidine levels and affects spermidine homeostasis via feedback involving translation control of several uORF-mRNAs which have roles in spermidine oxidation, such as *PAO* and *CuAO* (Salazar-Díaz *et al*., 2021). So far, it is largely unknown how many uORF-mRNAs are under TOR control, and how TOR selectively regulates translation of certain types of uORF-mRNAs, since the effect of TOR may vary from case to case as well as be modulated by the number, length, and nature of the uORFs.